# Hyperbaric Oxygen Therapy in Cancer: Friend, Foe, or Context-Dependent Modulator?

**DOI:** 10.3390/biology15171512

**Published:** 2026-09-03

**Authors:** Turan Demircan, Serkan Ergözen, Berna Yıldırım, Halil İbrahim Ünsal, Ayhan Bilir

**Affiliations:** 1 Medical Biology Department, School of Medicine, Izmir Bakircay University, Izmir 35665, Türkiye; turandemircan@gmail.com; 2 Underwater and Hyperbaric Medicine Department, School of Medicine, Mugla Sitki Kocman University, Mugla 48000, Türkiye; serkanergozen@mu.edu.tr; 3 Department of Histology and Embryology, Faculty of Medicine, Istanbul Atlas University, Istanbul 34403, Türkiye; berna.yildirim@atlas.edu.tr; 4 Department of Family Medicine, Provincial Directorate of Health, Diyarbakir 21120, Türkiye; halilibrahimunsal@hotmail.com

**Keywords:** hyperbaric oxygen therapy, tumor hypoxia, tumor microenvironment, hypoxia-inducible factors, redox homeostasis, cancer metabolism, treatment resistance, radiosensitization, radiation injury

## Abstract

Hyperbaric oxygen therapy (HBOT) increases tissue oxygen availability through the administration of near-pure oxygen under elevated atmospheric pressure. Although HBOT is clinically established for several hypoxia-related conditions and selected radiation-induced tissue injuries, its role in oncology remains controversial. This review evaluates HBOT as a context-dependent modulator of tumor and normal-tissue biology by integrating evidence on hypoxia-inducible factor signaling, redox homeostasis, tumor metabolism, vascular remodeling, immune regulation, extracellular matrix organization, cancer stemness, and treatment sensitivity. Available preclinical and clinical evidence does not support the historical assumption that HBOT universally accelerates tumor growth, recurrence, or metastasis, but current evidence is also insufficient to support HBOT as a broadly applicable standalone anticancer therapy. The strongest clinical evidence concerns late radiation-induced normal-tissue injury, particularly radiation cystitis, whereas evidence for direct antitumor activity or treatment sensitization remains predominantly preclinical and context-dependent. Selected experimental models suggest that transient reoxygenation may attenuate hypoxia-dependent signaling, alter redox and metabolic adaptation, and enhance sensitivity to radiotherapy or systemic anticancer treatment, while other models demonstrate increased proliferation or vascular responses. Future studies should incorporate standardized HBOT protocols, direct or surrogate measurements of tumor oxygenation, mechanistic biomarkers, patient stratification based on measurable tumor characteristics such as baseline hypoxia, redox phenotype, and microenvironmental features, and precise treatment scheduling. HBOT may ultimately be most relevant as a biomarker- and timing-guided pharmacodynamic reoxygenation strategy in tumors in which a transient reoxygenation window can be therapeutically exploited.

## Simple Summary

Hyperbaric oxygen therapy increases tissue oxygenation by allowing patients to breathe nearly pure oxygen under increased atmospheric pressure. It is already used to treat several conditions associated with poor tissue oxygenation and selected complications of radiotherapy. Its role in cancer remains controversial because increased oxygen availability may support normal-tissue repair, whereas tumor responses to reoxygenation are more variable. Tumor hypoxia can promote treatment resistance, metabolic adaptation, immune suppression, and aggressive tumor behavior. This review examines whether hyperbaric oxygen therapy should be considered beneficial, harmful, or dependent on biological and treatment context. Current evidence does not support the view that it universally accelerates tumor growth, recurrence, or metastasis, but it also does not justify its use as a standalone cancer treatment. The strongest clinical evidence supports its use for selected radiation-induced tissue injuries, whereas its potential as an anticancer adjunct remains largely investigational. Future studies should determine whether specific hypoxic tumors can be rendered more treatment-sensitive during a measurable and carefully timed period of reoxygenation.

## 1. Introduction

Hyperbaric oxygen therapy (HBOT) is a medical intervention in which a patient breathes medical-grade oxygen within a hyperbaric chamber at a pressure exceeding normal atmospheric pressure. The formal definition of HBOT should be distinguished from the specific pressure, oxygen concentration, and exposure duration selected for an individual treatment protocol. Clinical HBOT is generally defined by the administration of high-concentration oxygen within a pressurized chamber, whereas the exact therapeutic pressure and exposure schedule vary according to the clinical indication and protocol [1,2]. In oncological and radiation-injury studies, pressures of approximately 2.0–2.5 ATA are frequently used, but this range represents a commonly applied treatment protocol rather than the general definition of HBOT [2,3]. By increasing the amount of oxygen physically dissolved in plasma, HBOT can enhance tissue oxygen delivery beyond that provided by hemoglobin-bound oxygen alone [2,3]. This physiological effect underlies the established use of HBOT in conditions characterized by tissue hypoxia, impaired wound healing, ischemia, infection, gas embolism, and radiation-induced tissue injury [2,3,4]. Beyond these indications, hyperoxia can influence redox signaling, inflammatory responses, angiogenesis, mitochondrial function, immune activity, and cellular metabolism, making HBOT a biologically complex intervention whose effects depend strongly on tissue context [5,6,7,8].

This complexity is particularly relevant in cancer, where oxygen availability is a major determinant of tumor phenotype and therapeutic response. Chronic tumor hypoxia stabilizes hypoxia-inducible factors (HIFs) and promotes metabolic reprogramming, angiogenesis, epithelial–mesenchymal transition, cancer stemness, immune suppression, invasion, metastasis, and resistance to radiotherapy and systemic anticancer therapies [9,10,11,12]. Conversely, transient reoxygenation can perturb redox homeostasis, mitochondrial metabolism, vascular function, immune-cell activity, extracellular matrix organization, and susceptibility to treatment-induced damage [12,13,14,15,16,17]. HBOT should therefore not be regarded simply as an oxygen-supplementation strategy but rather as a transient perturbation of tumor oxygenation and redox homeostasis whose biological consequences are shaped by baseline hypoxia, vascular and stromal architecture, metabolic phenotype, antioxidant capacity, host-tissue condition, and treatment timing [12,13,14,16].

Historically, the use of HBOT in patients with cancer has been constrained by concerns that increased oxygen availability might stimulate tumor proliferation, angiogenesis, recurrence, or metastatic progression [18,19]. This concern is not purely theoretical, because reoxygenation of previously hypoxic tumor-cell populations can itself produce transient biological responses that may favor malignant behavior [18]. In an early experimental study, tumor cells isolated from hypoxic regions of murine solid tumors exhibited a transient increase in lung-colonizing ability after reoxygenation, and the most hypoxic subpopulations showed greater metastatic efficiency than cells derived from better-oxygenated regions [18]. These observations indicate that the transition from hypoxia to reoxygenation should not be assumed to be uniformly beneficial and may generate distinct biological responses depending on the tumor-cell population and microenvironmental context [18,19]. However, accumulated experimental and clinical evidence has not demonstrated a consistent tumor-promoting effect, challenging the assumption that HBOT is intrinsically unsafe in the oncological setting [3,20]. More recent studies have instead emphasized its potential to alleviate tumor hypoxia, modify the tumor microenvironment, improve therapeutic penetration, enhance radiation-induced cytotoxicity, alter redox signaling, and increase responsiveness to selected anticancer treatments [16,21]. Nevertheless, these effects are heterogeneous and cannot be generalized across tumor types, disease settings, treatment regimens, or HBOT protocols.

Accordingly, the central question is not simply whether HBOT is beneficial or harmful in cancer, but under which biological and therapeutic conditions reoxygenation produces adverse, neutral, or therapeutically exploitable effects. This review examines HBOT as a context-dependent modulator of tumor and normal-tissue biology, integrating evidence on HIF signaling, redox regulation, metabolism, vascular and immune responses, extracellular matrix organization, cancer stemness, and treatment resistance. Particular emphasis is placed on reconciling tumor-growth concerns with evidence that controlled reoxygenation may disrupt hypoxia-dependent resistance mechanisms or enhance selected anticancer therapies [16,18,19].

## 2. Oxygen as a Central Regulator of Energy Metabolism, Redox Homeostasis, and Tissue Function

Oxygen is indispensable for aerobic metabolism and is a central determinant of cellular and tissue homeostasis. At the mitochondrial level, molecular oxygen serves as the terminal electron acceptor in the electron transport chain, enabling oxidative phosphorylation and efficient adenosine triphosphate (ATP) generation. Although ATP can also be generated through glycolysis under oxygen-limited conditions, sustained cellular function in metabolically active tissues depends predominantly on mitochondrial oxidative metabolism [8,22].

Tissue oxygenation reflects the coordinated function of pulmonary gas exchange, cardiovascular perfusion, and hemoglobin-mediated oxygen transport. Following pulmonary uptake, oxygen diffuses across the alveolar–capillary membrane and binds reversibly to hemoglobin before being delivered to peripheral tissues. Oxygen unloading is dynamically influenced by local oxygen tension, carbon dioxide concentration, pH, temperature, and 2,3-bisphosphoglycerate, allowing oxygen delivery to adapt to regional metabolic demand [23]. HBOT modifies this physiological relationship by markedly increasing the amount of oxygen physically dissolved in plasma, thereby augmenting oxygen delivery to hypoxic tissues beyond that achievable through hemoglobin-bound transport alone [2,3].

**Figure 1:**
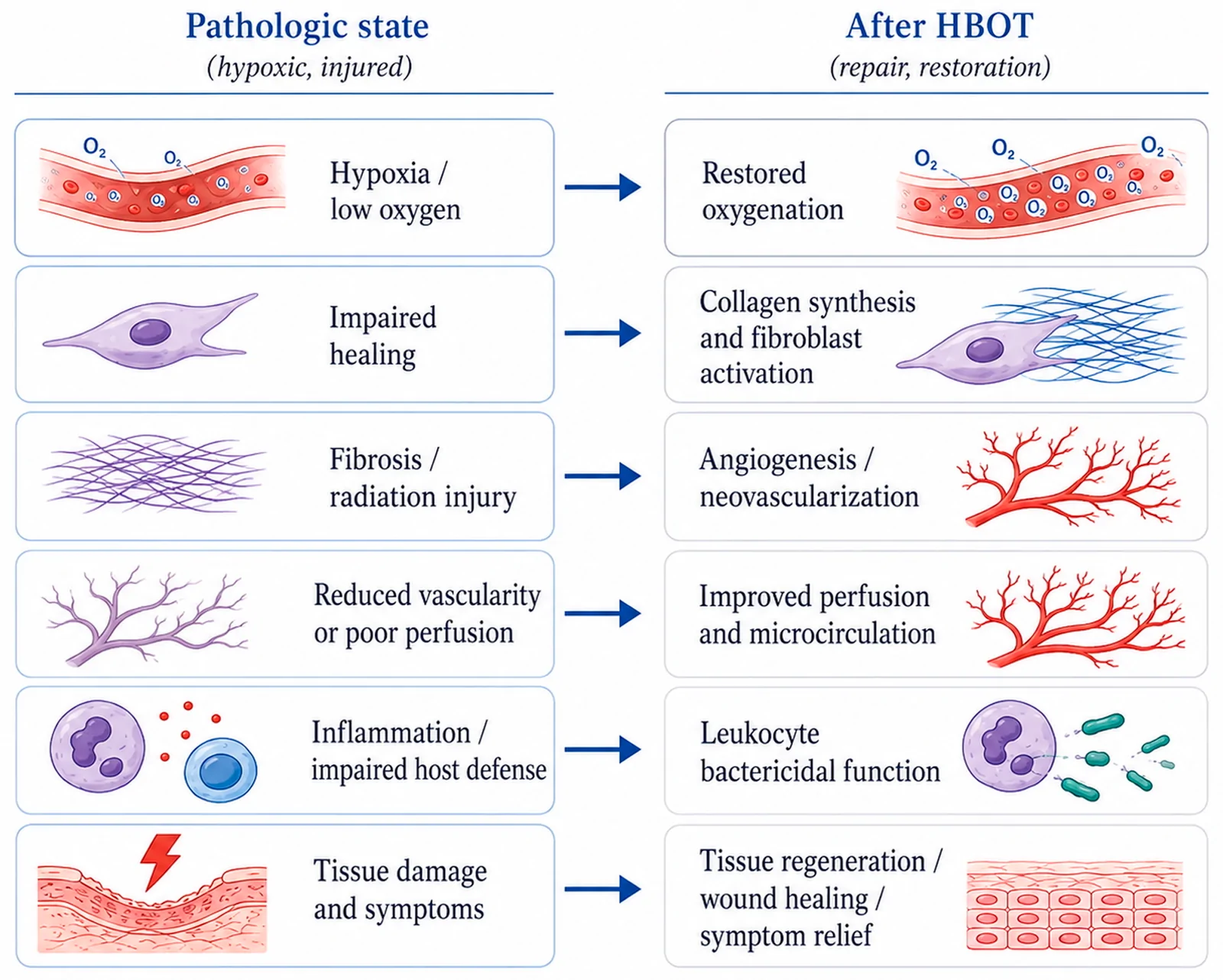
HBOT-mediated restoration of pathological tissue. Schematic representation of the major consequences of hypoxia or tissue injury and their modulation by hyperbaric oxygen therapy. By increasing tissue oxygen availability, HBOT may support fibroblast activation and collagen synthesis, promote angiogenesis and microvascular remodeling, improve tissue perfusion, enhance oxygen-dependent leukocyte bactericidal activity, and facilitate epithelial and connective-tissue regeneration. Collectively, these processes contribute to wound healing and functional recovery in hypoxic, ischemic, or radiation-injured normal tissues. (Created in BioRender).

Beyond its role in energy metabolism, oxygen is a key regulator of redox signaling, immune defense, vascular adaptation, and tissue repair. Reactive oxygen species (ROS), generated by mitochondria and enzymatic systems such as NADPH oxidases, are not merely toxic metabolic by-products but also function as signaling intermediates. At controlled concentrations, ROS participate in the regulation of proliferation, differentiation, inflammatory responses, stress adaptation, and cell-survival pathways [7,8]. Oxygen-dependent oxidative mechanisms are also essential for innate immune defense, particularly the respiratory burst used by neutrophils and macrophages for microbial killing. In injured tissues, adequate oxygen availability supports fibroblast activity, collagen synthesis, reparative angiogenesis, extracellular matrix remodeling, epithelial repair, and other processes required for tissue regeneration and wound healing [6,24]. These interconnected oxygen-dependent processes and their modulation by HBOT in hypoxic or injured normal tissues are summarized in Figure 1.

Cellular adaptation to oxygen availability is tightly regulated by oxygen-sensing pathways, among which hypoxia-inducible factors (HIFs) play a central role. Under hypoxic conditions, HIF stabilization activates transcriptional programs that promote angiogenesis, erythropoiesis, glycolytic metabolism, cellular survival, and adaptation to reduced oxygen availability [25]. Although these responses are physiologically protective during transient oxygen deprivation, persistent HIF activation within the tumor microenvironment can support metabolic reprogramming, angiogenesis, malignant progression, and resistance to anticancer therapy [9,12]. Reoxygenation can therefore influence not only tissue oxygen tension but also the signaling networks that govern cellular adaptation to hypoxia [19,25].

The biological effects of oxygen are dose-, duration-, and context-dependent. Excessive or prolonged oxygen exposure can increase oxidative stress and promote lipid, protein, DNA, and mitochondrial damage, whereas controlled increases in reactive oxygen species may function as adaptive signaling events [8,26]. This context dependence is particularly relevant in oncology, where constitutive oxidative stress, altered metabolism, hypoxia-dependent signaling, and adaptive antioxidant systems can substantially modify the response to HBOT-induced reoxygenation [12,14].

## 3. HBOT at the Interface of Repair, Redox Biology, and Microenvironment Modulation

The oncological controversy surrounding hyperbaric oxygen therapy (HBOT) reflects the fundamentally different consequences of oxygenation in injured normal tissues and malignant tissues. In ischemic or radiation-injured normal tissues, improved oxygen availability supports reparative processes such as collagen synthesis, fibroblast activity, leukocyte function, angiogenesis, and tissue regeneration [6,27]. In tumors, however, oxygen availability interacts with a microenvironment shaped by chronic hypoxia, altered metabolism, aberrant vasculature, oxidative stress, and therapeutic selection pressures. Chronic tumor hypoxia stabilizes HIF-1α and HIF-2α, promotes VEGF-dependent angiogenic signaling and glycolytic reprogramming, facilitates epithelial–mesenchymal transition and stem-like phenotypes, and contributes to immune suppression and treatment resistance [9,11,12]. Reoxygenation, therefore, cannot be assumed to reproduce the reparative effects observed in normal tissues; depending on tumor phenotype and treatment context, it may instead disrupt hypoxia-dependent adaptations or generate new biological responses [12,16,19].

Importantly, reoxygenation itself may generate biologically unfavorable responses in selected tumor-cell populations. Cells residing in chronically or intermittently hypoxic regions of solid tumors are exposed to substantial metabolic and selective pressures, and restoration of oxygen availability does not necessarily return these cells immediately to the phenotype of continuously well-oxygenated tumor cells. Experimental evidence has shown that tumor cells isolated from hypoxic regions can display a transient increase in metastatic colonization after reoxygenation, suggesting that hypoxia–reoxygenation transitions may temporarily favor phenotypes associated with metastatic competence [18]. Reoxygenation may also alter reactive oxygen species production, cell-cycle regulation, metabolic activity, and oxygen-sensitive signaling pathways, generating outcomes that differ according to the severity and duration of preceding hypoxia and the adaptive state of the tumor cells [19]. Thus, chronic hypoxia, acute reoxygenation, and sustained normoxia should be regarded as biologically distinct states rather than interchangeable levels of oxygen availability. This distinction is particularly relevant to HBOT, which produces an intermittent and temporally defined perturbation of tissue oxygenation rather than permanent normalization of the tumor microenvironment [18,19].

One of the strongest mechanistic rationales for integrating HBOT with cancer therapy is radiosensitization. According to the oxygen fixation hypothesis, molecular oxygen reacts with radiation-induced DNA radicals, increasing the probability that potentially reversible lesions become chemically fixed and biologically lethal [28]. Under hypoxic conditions, radiation-induced radicals are more readily restored before permanent damage is established, contributing to radioresistance [29]. Transient elevation of oxygen tension in hypoxic tumor regions may therefore increase the biological effectiveness of radiotherapy. However, this potential benefit is expected to depend strongly on treatment scheduling because the therapeutic value of reoxygenation is greatest when irradiation coincides with the period of increased tumor oxygen availability [28,29].

Vascular responses provide another example of why HBOT cannot be interpreted uniformly across normal and malignant tissues. In ischemic or injured normal tissues, hyperoxia can support repair-associated angiogenesis, collagen deposition, tissue perfusion, and endothelial responses [6,27,30]. Within tumors, however, increased vascularity is not necessarily equivalent to tumor-promoting angiogenesis. Chronic hypoxia itself drives HIF–VEGF signaling and contributes to structurally abnormal and poorly functional tumor vessels. Reoxygenation may alter this proangiogenic signaling environment and, in selected settings, contribute to vascular remodeling or normalization, with potential effects on perfusion, interstitial pressure, therapeutic delivery, and immune-cell trafficking [13,21]. Consequently, changes in individual vascular markers should not be interpreted in isolation as evidence of tumor stimulation; they should be considered together with tumor oxygenation, perfusion, growth kinetics, and treatment response [13,21].

Redox modulation represents a second major component of HBOT biology. Hyperoxia increases the generation of reactive oxygen and nitrogen species and can alter intracellular redox signaling [26,31]. The biological consequences of this perturbation depend on exposure magnitude and duration, mitochondrial function, antioxidant capacity, and the baseline oxidative state of the tissue. In normal tissues, controlled ROS signaling can participate in wound repair, inflammatory regulation, fibroblast responses, and vascular adaptation [7,24]. Cancer cells, in contrast, frequently exist under chronically elevated oxidative stress generated by oncogenic signaling, mitochondrial dysfunction, rapid proliferation, inflammation, and recurrent hypoxia–reoxygenation cycles. Many tumors compensate by increasing antioxidant capacity through NRF2-dependent signaling, glutathione and thioredoxin systems, catalase, superoxide dismutase, and metabolic redox buffering [12,14]. HBOT-induced oxidative stress may therefore exceed the buffering capacity of selected tumor cells, whereas tumors with robust antioxidant defenses may tolerate transient hyperoxia more effectively. Baseline redox phenotype may thus be an important determinant of HBOT response.

Hypoxia also contributes to immune suppression within the tumor microenvironment. HIF-dependent signaling can promote immunosuppressive myeloid phenotypes, enhance extracellular adenosine signaling through the CD39/CD73 axis, and impair cytotoxic T-cell activity [15]. Reoxygenation may therefore influence antitumor immunity indirectly by attenuating selected components of hypoxia-associated immune suppression. Emerging preclinical evidence suggests that HBOT can modify immune-cell infiltration, stromal organization, and responses to immune checkpoint blockade in selected experimental settings [16]. These observations remain preliminary, but they support the concept that the consequences of HBOT extend beyond oxygen tension itself to the immunometabolic organization of the tumor microenvironment.

Extracellular matrix remodeling provides an additional interface between hypoxia, vascular dysfunction, and treatment resistance. Hypoxic tumors frequently exhibit increased collagen deposition, matrix stiffening, lysyl oxidase activity, elevated interstitial pressure, and impaired perfusion, thereby creating physical and biochemical barriers to immune-cell trafficking and therapeutic penetration [17]. By modifying hypoxia-dependent stromal signaling and vascular–matrix interactions, HBOT has been proposed as a means of improving therapeutic penetration, particularly in combination with immunotherapy or drug-delivery platforms [16,21]. This possibility may be particularly relevant in tumors characterized by profound hypoxia, dense stroma, and poor perfusion; however, direct evidence that HBOT reproducibly remodels these barriers in patients remains limited.

**Figure 2:**
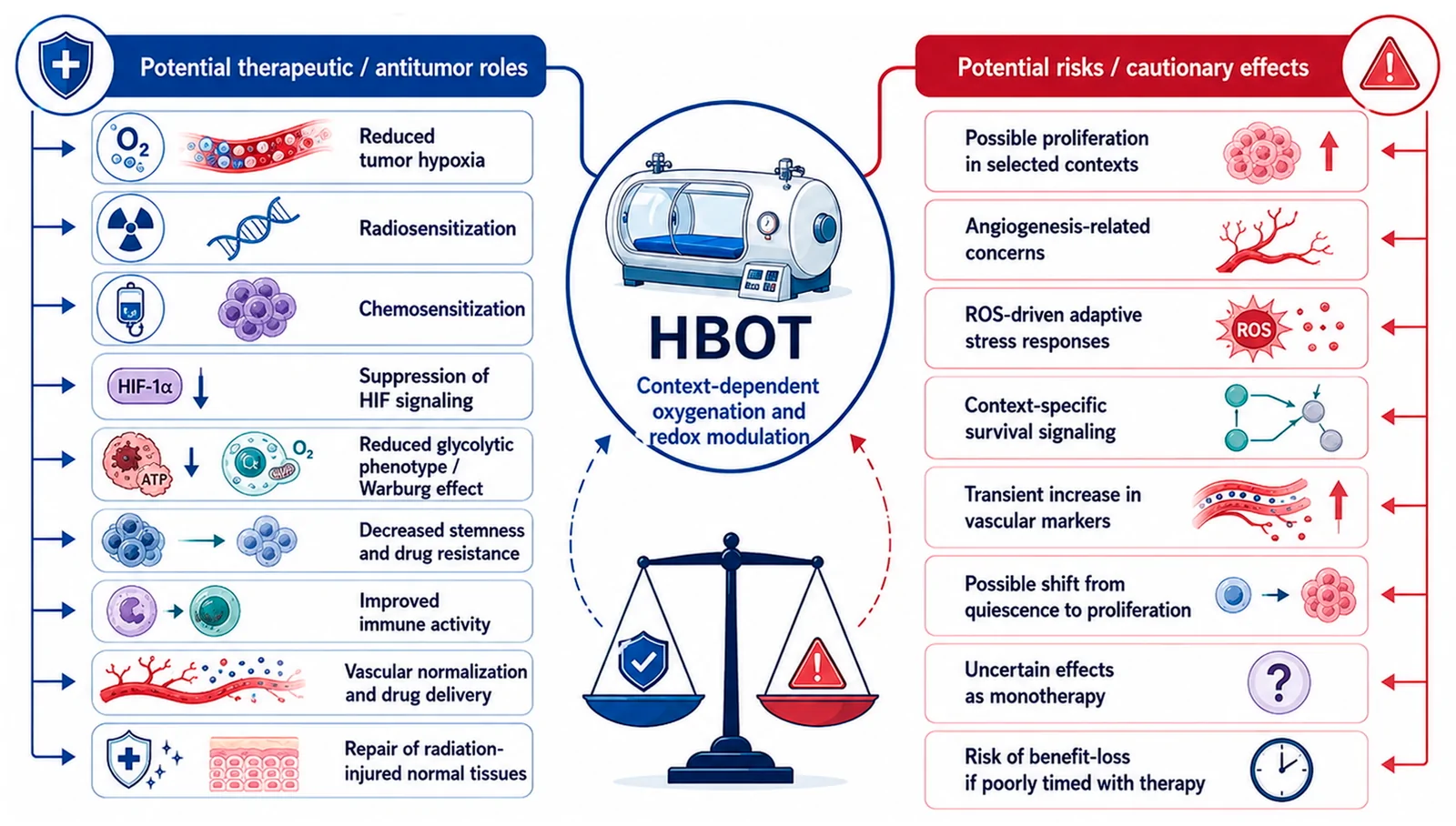
Context-dependent biological consequences of HBOT in oncology. Schematic overview of potential therapeutic and cautionary responses to hyperbaric oxygen therapy. In selected tumor contexts, HBOT may reduce hypoxia, enhance radiosensitivity or chemosensitivity, suppress HIF-dependent signaling, alter glycolytic metabolism, reduce stemness, modulate immune activity, and influence vascular function. In injured normal tissues, HBOT may independently support repair after radiation injury. Conversely, selected tumor settings may exhibit increased proliferation, vascular-marker changes, adaptive redox signaling, or loss of therapeutic benefit when reoxygenation is poorly synchronized with anticancer treatment. The net biological response depends on tumor phenotype, baseline hypoxia, redox capacity, microenvironmental organization, and treatment timing (Created in BioRender).

Taken together, HBOT cannot be characterized as uniformly tumor-promoting or tumor-suppressive. Its effects depend on tumor phenotype, baseline hypoxia, redox capacity, microenvironmental organization, and treatment timing. Its most plausible oncological role is therefore as a controlled adjunct intended to exploit a biologically defined period of reoxygenation rather than as a standalone anticancer intervention [3,16,19]. These context-dependent therapeutic and cautionary effects of HBOT in oncology are summarized in Figure 2.

## 4. HBOT and Tumor Growth: Reassessing a Long-Standing Oncological Concern

Concerns that HBOT may accelerate tumor growth have persisted because oxygen availability and vascular signaling are integral to tumor biology. Nevertheless, the available experimental and clinical literature does not support HBOT as a general promoter of tumor growth, recurrence, or metastasis, although the heterogeneous evidence base precludes universal safety conclusions [3,20].

An additional layer of complexity is that reduced oxygen availability is not uniformly growth-promoting and may itself impose important constraints on tumor expansion. Severe or sustained oxygen limitation can restrict oxidative metabolism and biosynthetic activity, limit the proliferative capacity of tumor cells, and contribute to cell-cycle arrest, quiescence, or cell death when adaptive mechanisms are insufficient. At the same time, hypoxia can select for tumor-cell populations capable of metabolic adaptation, survival under stress, invasion, and resistance to anticancer therapy, explaining why localized intratumoral hypoxia is generally associated with treatment resistance and an adverse tumor phenotype [19,32]. This apparent paradox has been further highlighted by recent preclinical evidence showing that systemic hypoxia, in contrast to localized intratumoral hypoxia, can suppress solid-tumor growth across multiple experimental cancer models [33]. In that study, systemic hypoxia was associated with perturbation of purine metabolism and suppression of de novo purine synthesis, suggesting that oxygen restriction can impose a metabolic limitation on tumor growth under specific conditions [33]. Importantly, these findings derive from a preprint that has not yet undergone peer review, and systemic hypoxia is biologically distinct from the spatially heterogeneous hypoxia that develops within poorly perfused regions of solid tumors. Therefore, the observation that systemic hypoxia can restrict tumor growth should not be interpreted as evidence that localized tumor hypoxia is generally protective; rather, it emphasizes that oxygen availability can simultaneously impose metabolic constraints on tumor biomass and create selective pressures favoring malignant adaptation, depending on its severity, duration, spatial distribution, and physiological context [19,32,33].

Experimental findings should therefore be interpreted according to tumor type, model system, oxygen exposure, baseline hypoxia, and concurrent treatment. Across colorectal, prostate, breast, and glioma models, responses range from absence of tumor acceleration or suppression of hypoxia-associated phenotypes to increased proliferation or vascular-marker expression in selected settings [20,34,35,36,37]. The following sections examine these tumor-specific differences and their implications for treatment sensitivity and supportive care.

### 4.1. HBOT Does Not Accelerate Prostate Tumor Growth in Preclinical Models

Available prostate cancer models generally do not support the hypothesis that hyperbaric oxygen therapy (HBOT) accelerates tumor growth. In murine xenograft studies using LNCaP and PC-3 prostate cancer cells, repeated HBOT exposure did not produce sustained increases in tumor growth or angiogenic activity compared with control conditions [34,38]. Importantly, a separate latent prostate cancer model likewise found no evidence that HBOT accelerated tumor progression, an observation of particular relevance to the use of HBOT for radiation-induced hemorrhagic cystitis in prostate cancer survivors [39].

In vitro findings indicate a more complex biological response. Hyperbaric oxygen reduced the growth rate of DU-145 prostate cancer cells and produced more modest effects on PC-3 cells, while also altering chemosensitivity in a drug-dependent manner. Increased sensitivity was reported for paclitaxel and, to a lesser extent, doxorubicin, whereas cisplatin responsiveness was not substantially changed [40]. Hyperbaric oxygen exposure has also been associated with cell-cycle perturbation in LNCaP cells, including increased DNA synthesis and accumulation in the G2/M phase [41]. Such changes should not be interpreted in isolation as evidence of tumor promotion, because altered cell-cycle distribution may also modify susceptibility to cytotoxic treatment.

Prostate cancer models therefore do not demonstrate a consistent tumor-promoting effect of HBOT, although the evidence remains predominantly preclinical and insufficient to establish an anticancer role [34,38,39,40,41].

### 4.2. HBOT and the VHL–HIF Axis in Clear Cell Renal Cell Carcinoma

Clear cell renal cell carcinoma (ccRCC) provides a particularly relevant model for considering the relationship between oxygen availability and HIF signaling because its molecular pathogenesis frequently involves loss or inactivation of the von Hippel–Lindau (VHL) tumor-suppressor gene. Under normal oxygen-sensing conditions, pVHL participates in the proteasomal degradation of HIF-α subunits. Loss of functional VHL therefore results in abnormal stabilization of HIF signaling, particularly HIF-2α, and activation of transcriptional programs involved in angiogenesis, metabolic reprogramming, cell survival, and tumor progression even when oxygen availability is not severely limited. This constitutive or “pseudohypoxic” phenotype is a defining feature of a substantial proportion of ccRCCs and provides the biological rationale for therapies directed against the HIF-2α pathway [42,43].

This biology also has important implications for the potential effects of HBOT. In tumors in which HIF stabilization is primarily driven by microenvironmental oxygen deprivation, reoxygenation may attenuate oxygen-sensitive HIF signaling. In VHL-deficient ccRCC, however, increased oxygen availability alone may be insufficient to normalize HIF-2α activity because the molecular machinery responsible for oxygen-dependent HIF degradation is disrupted. Accordingly, the effects of HBOT in ccRCC cannot be predicted solely from its ability to increase tumor oxygen tension. Direct experimental evidence evaluating HBOT as an antitumor intervention in VHL-deficient ccRCC remains very limited, and no established therapeutic role can currently be inferred. ccRCC therefore represents an important example in which the genetic basis of pseudohypoxia may determine whether reoxygenation meaningfully alters tumor biology. Future studies should directly evaluate whether HBOT modifies HIF-independent redox, metabolic, vascular, or immune pathways in ccRCC and whether VHL status influences the biological response to transient reoxygenation [42,43].

### 4.3. HBOT Suppresses Hypoxia-Driven Progression in Lung Cancer Models

In lung cancer models, current experimental evidence suggests that hyperbaric oxygen therapy (HBOT) can counteract selected malignant phenotypes associated with hypoxia-dependent adaptation. In an A549 xenograft model, HBOT improved tumor oxygenation and reduced tumor growth while increasing CD31-positive vascular structures and cleaved caspase-3-associated apoptosis [44]. This finding is notable because increased vascular-marker expression occurred in parallel with tumor suppression, illustrating that isolated changes in vascular markers do not necessarily indicate tumor-promoting angiogenesis.

Mechanistic studies in non-small cell lung cancer (NSCLC) further support a role for hypoxia signaling and metabolic reprogramming in the response to oxygenation. In hypoxic NSCLC cells, HBOT inhibited HIF-1α/PFKP signaling and attenuated glycolytic metabolism, hyperproliferation, and epithelial–mesenchymal transition [45]. Additional experiments in A549 and H1299 cells showed that hyperbaric oxygen altered hypoxia-associated glucose metabolism, including glucose uptake, lactate production, pyruvate levels, and ATP-related metabolic responses; HIF-1α overexpression attenuated these effects, supporting a role for HIF-1α-dependent metabolic regulation [46].

Chemically induced hypoxia models provide complementary evidence. In CoCl_2_-treated A549 cells, HBOT attenuated several hypoxia-associated malignant features, including migration, invasion, epithelial–mesenchymal transition-related changes, apoptosis resistance, and alterations in the Bcl-2/Bax axis and GRP78 expression [47]. These observations suggest that the effects of reoxygenation extend across multiple components of the hypoxia-adaptive phenotype rather than being mediated through a single pathway.

Evidence from oxygen-based interventions other than HBOT provides additional mechanistic context, although such findings should not be considered direct evidence for hyperbaric treatment. In experimental lung cancer models, inspiratory hyperoxia reduced metastatic progression and improved survival through mechanisms involving MYC/SLC1A5-dependent metabolic regulation and glutamine utilization [48]. These findings support the broader biological principle that increased oxygen availability can disrupt metabolic dependencies created by chronic hypoxia, while remaining mechanistically distinct from HBOT.

Taken together, preclinical lung cancer models suggest that HBOT can disrupt selected hypoxia-associated metabolic and invasive phenotypes, but clinical anticancer efficacy has not been established [44,45,46,47].

### 4.4. HBOT Enhances Anticancer Responses in Experimental Colorectal Cancer Models

In colorectal cancer, the available experimental evidence does not support the view that hyperbaric oxygen therapy (HBOT) intrinsically promotes tumor growth. In a murine model of colorectal cancer liver metastasis, daily HBOT did not increase metastatic tumor volume or produce evidence of adverse microvascular progression [49]. Although transient changes in tumor necrosis and proliferative activity were observed at early time points, these effects were not sustained, arguing against persistent tumor acceleration in this model.

The potential value of HBOT appears more evident when it is combined with anticancer treatment. In chemically induced experimental colorectal neoplasia, HBOT combined with 5-fluorouracil increased apoptosis within dysplastic crypts and reduced expression of the hypoxia- and stemness-associated markers HIF-1α and CD44 compared with chemotherapy alone [50]. These findings are consistent with the possibility that reoxygenation can weaken hypoxia-associated survival or stemness programs and thereby increase susceptibility to cytotoxic therapy.

Combination strategies involving drug-delivery systems have also been explored. In a colorectal liver-metastasis model, SMA–pirarubicin micelles showed greater antitumor activity than free pirarubicin, and the addition of HBOT was associated with further therapeutic benefit [51]. Increased oxygen availability may have contributed to this interaction by modifying the redox environment in which anthracycline-induced damage occurs; however, this mechanistic explanation remains inferential and should not be regarded as established by the study itself.

Additional mechanistic evidence suggests that HBOT can influence multiple pathways implicated in colorectal tumor progression. In an experimental colorectal carcinogenesis model, HBOT alone or in combination with melatonin was associated with increased apoptosis, reduced tumor proliferation, decreased HIF-1α expression, attenuation of AKT and glycolytic signaling, suppression of stemness-related pathways, and reversal of epithelial–mesenchymal transition-associated changes [52]. These pleiotropic effects are consistent with the broader concept that transient reoxygenation can interfere with several adaptive programs maintained by chronic tumor hypoxia.

Current colorectal cancer studies show no consistent tumor-promoting effect and suggest potential adjunctive activity with selected treatments; however, the evidence remains preclinical [20,50,51,52].

### 4.5. HBOT in Breast Cancer-Related Radiation Injury and Oxygen-Dependent Tumor Biology

In breast cancer, the clinical literature on hyperbaric oxygen therapy (HBOT) primarily concerns the management of late radiation-induced tissue toxicity rather than direct antitumor treatment. Observational and prospective studies have reported improvements in symptoms including breast and arm pain, swelling, hypersensitivity, shoulder dysfunction, fibrosis, and overall symptom burden following HBOT. In a large Dutch cohort of patients with late radiation toxicity after breast cancer treatment, HBOT was associated with reductions in pain, breast symptoms, and arm symptoms, together with improvements in several quality-of-life domains; treatment-related adverse effects, including transient myopia and mild barotrauma, were generally reversible [53]. Prospective patient-reported outcome data have similarly suggested improvements in pain, swelling, skin-related symptoms, and functional complaints [54].

Evidence synthesis supports a potential clinical benefit while also emphasizing important limitations in the underlying literature. A systematic review including more than 1300 patients concluded that HBOT may reduce manifestations of local late radiation toxicity, particularly pain, fibrosis, lymphedema, and mobility impairment, but noted substantial heterogeneity, small study populations, non-randomized designs, and an overall risk of bias [55]. Randomized evidence provides a more cautious interpretation. In women with late local toxicity following breast irradiation, assignment to an HBOT treatment strategy did not significantly reduce pain in the intention-to-treat analysis, in part because only a minority of eligible patients initiated and completed treatment. Fibrosis, however, was significantly reduced, and patients who completed HBOT experienced improvements in both pain and fibrosis [56]. Small studies focused specifically on breast cancer-related arm lymphedema have suggested possible reductions in limb volume or improvements in tissue characteristics, although the limited sample sizes and control data preclude firm conclusions [57].

Beyond supportive care, preclinical evidence indicates that HBOT does not necessarily promote breast tumor progression and may exert antitumor effects in selected experimental models. In xenograft models using MDA-MB-231 triple-negative breast cancer cells and BT-474 breast cancer cells, HBOT suppressed tumor growth and reduced proliferative activity [35]. In the MDA-MB-231 model, HBOT was also associated with reduced metastatic burden and decreased expression of epithelial–mesenchymal transition-associated markers, including N-cadherin and Axl. However, HBOT did not enhance the effect of 5-fluorouracil in the same experimental setting, indicating that interactions between reoxygenation and chemotherapy are likely to depend on both tumor phenotype and treatment regimen [35].

Direct anticancer evidence in breast cancer remains limited. Although experimental xenografts suggest that HBOT can reduce tumor growth and metastatic features in selected models, clinical evidence primarily supports its use for late radiation toxicity rather than tumor treatment [35]. A multimodal case report combining HBOT with chemotherapy, ketogenic diet, and hyperthermia cannot isolate an HBOT-specific effect and should be considered hypothesis-generating [58].

### 4.6. HBOT in Experimental Melanoma Models

Melanoma provides an additional model in which the effects of HBOT may be relevant to both tumor-cell behavior and the organization of the tumor microenvironment. Direct experimental evidence remains limited. In an early study using B16 melanoma transplanted into C57BL mice, hyperbaric oxygen exposure did not stimulate primary tumor growth or metastatic dissemination. Across the experimental tumor models examined in that study, HBO produced no consistent tumor-promoting effect and was associated with a modest inhibitory tendency in some settings, although the age and design of the study limit conclusions regarding contemporary melanoma biology or therapeutic combinations [59].

More recent work has demonstrated that melanoma oxygenation can respond markedly to hyperbaric exposure. Real-time in vivo EPR oximetry in B16-F10 melanoma showed an increase in tumor oxygen tension from approximately 9 mmHg at baseline to approximately 26 mmHg during 100% oxygen at normobaric pressure and to approximately 50 mmHg during 100% oxygen at 2 ATA, with partial persistence of the oxygenation effect after decompression. Importantly, a different solid-tumor model exposed to the same conditions showed minimal oxygenation response, indicating substantial tumor-specific heterogeneity in the delivery of oxygen under hyperbaric conditions [60]. These observations demonstrate that HBOT can substantially modify the hypoxic state of at least some melanoma models, but they do not establish an antitumor effect [60]. Given the highly interactive immune, vascular, and stromal microenvironment of melanoma, future studies should determine whether such changes in oxygenation modify immune-cell infiltration, inflammatory signaling, metastatic behavior, or responsiveness to immune checkpoint blockade.

### 4.7. The Dual Role of HBOT in Glioblastoma and Glioma

Glioblastoma and glioma provide one of the clearest examples of the context-dependent biological effects of hyperbaric oxygen therapy (HBOT), because the available evidence includes both treatment-sensitizing and potentially proliferation-promoting responses. Tumor hypoxia is a central feature of glioblastoma biology and contributes to radioresistance, chemoresistance, cancer stemness, invasive behavior, and recurrence. HBOT has therefore been investigated primarily as an adjunctive strategy intended to transiently increase tumor oxygenation and weaken hypoxia-dependent resistance mechanisms [61,62].

Experimental and translational studies suggest that increased oxygen availability can enhance treatment sensitivity through effects on HIF signaling, redox state, metabolism, and stemness. HBOT has been reported to improve oxygenation of neoplastic tissue and enhance the biological effects of ionizing radiation when irradiation is delivered during or shortly after reoxygenation [61,62]. In both in vitro and in vivo glioblastoma models, HBOT has also been associated with reduced cancer stem-cell formation and increased sensitivity to temozolomide and radiotherapy [63].

Evidence from patient-derived glioblastoma cells further supports a treatment-sensitizing effect of hyperoxic exposure. High-pressure oxygen inhibited proliferation, downregulated HIF-1α, altered glucose metabolism, increased intracellular reactive oxygen species, activated inflammasome-associated signaling, and enhanced radiosensitivity in two-dimensional and three-dimensional culture systems [64]. Notably, the same study reported relative radioprotection in microglial cells, indicating that malignant and non-malignant components of the brain microenvironment may respond differently to hyperoxic exposure.

HBOT may also influence the immune compartment of the glioblastoma microenvironment. In experimental models, HBOT has been associated with a shift in macrophage polarization away from an M2-like tumor-supportive phenotype toward a more M1-like phenotype, accompanied by reduced glioblastoma-cell viability and increased apoptosis [65]. These findings suggest that the consequences of reoxygenation may extend beyond tumor-cell intrinsic pathways to include immune and stromal components of the glioblastoma microenvironment.

Clinical evidence remains limited and should be interpreted cautiously. Reviews of glioma and brain-metastasis studies have described potential roles for HBOT in radiosensitization and multimodal treatment strategies, but the available clinical literature is small and heterogeneous [66,67]. In one clinical study, intensity-modulated radiotherapy boosts delivered immediately after HBOT in combination with chemotherapy were reported to be feasible and associated with mild toxicity; median overall survival was 22.1 months, with 2-year overall and progression-free survival rates of 46.5% and 35.4%, respectively [68]. These outcomes cannot establish an independent therapeutic effect of HBOT because of the multimodal treatment design, limited sample size, and absence of definitive comparative evidence.

Importantly, HBOT does not produce uniformly antitumor effects in glioma models. In an intracranial GL261 model, HBOT increased tumor growth, Ki-67 and CD34 expression, and microvessel density while reducing apoptosis [36]. Other studies demonstrated increased glioblastoma-cell proliferation alongside suppression of HIF-1α/HIF-2α–Sox2 signaling, reduced stemness, and enhanced chemosensitivity [37,69]. These findings illustrate that reoxygenation may simultaneously release hypoxia-imposed constraints on proliferation and reduce hypoxia-dependent stemness or treatment resistance [36,37,69].

### 4.8. HBOT for Radiation-Induced Tissue Injury in Oncological Care

In several oncological settings, hyperbaric oxygen therapy (HBOT) has been investigated primarily as a supportive treatment for radiation-induced tissue injury rather than as a direct anticancer intervention. This distinction is particularly evident after pelvic radiotherapy, where radiation-induced hemorrhagic cystitis represents one of the most extensively studied HBOT indications in cancer survivors. Chronically irradiated tissues are characterized by impaired perfusion, hypoxia, fibrosis, and defective tissue repair; by increasing tissue oxygen availability, HBOT can support neovascularization, fibroblast activity, epithelial repair, and restoration of tissue integrity [70]. This supportive-care rationale should be distinguished from the separate and considerably less established hypothesis that transient tumor reoxygenation might enhance sensitivity to anticancer therapy.

Clinical evidence is strongest for radiation-induced hemorrhagic cystitis following pelvic radiotherapy. Retrospective studies have reported complete or partial resolution of hematuria after HBOT in patients previously treated for prostate, bladder, cervical, and endometrial cancers [71]. Evidence syntheses have reported similarly favorable outcomes. A scoping review and meta-analysis including 602 patients found partial or complete resolution of hemorrhagic radiation cystitis in 84% of treated patients, with recurrence reported in 14% of patients for whom follow-up data were available [72]. A subsequent systematic review and meta-analysis including 556 patients reported symptomatic improvement in approximately 90% of cases and complete remission of hematuria in 55% [73]. Although differences in patient selection, HBOT protocols, outcome definitions, and study design limit direct comparison across studies, these findings support a clinically meaningful role for HBOT in selected patients with radiation-induced hemorrhagic cystitis.

Randomized evidence further strengthens the clinical basis for this indication. The RICH-ART trial and its long-term follow-up demonstrated sustained improvement in patient-reported urinary symptoms following HBOT for late radiation cystitis, with treatment generally being well tolerated [74,75]. Multicenter registry data have likewise shown improvements in hematuria, urinary distress, and patient-reported quality-of-life outcomes following HBOT [76]. Together, these findings provide a substantially stronger clinical evidence base for the treatment of radiation injury than currently exists for the use of HBOT as a direct anticancer intervention.

Smaller clinical series and case reports provide additional supportive observations but contribute a lower level of evidence. HBOT has been associated with control of severe radiation-induced hematuria in medically complex patients [77]. In a retrospective single-center study of radiation-induced hemorrhagic cystitis and proctitis, HBOT was associated with reductions in LENT-SOMA scores; among evaluable patients with hemorrhagic cystitis, complete resolution of hematuria was reported in 81% and partial response in 18% [78]. Such findings are consistent with the broader clinical literature but should not substitute for randomized or prospectively controlled evidence.

Collectively, radiation cystitis represents the oncological setting with the strongest clinical evidence for HBOT [72,73,74,76]. This supportive-care indication should remain clearly distinguished from the predominantly preclinical investigation of HBOT as a direct or adjunctive anticancer intervention. The comparative evidence is summarized in Table 1.

## 5. Current Challenges and Future Directions

Translation of HBOT into clinical oncology is limited by substantial heterogeneity in treatment pressure, exposure duration, session number, treatment frequency, and the interval between HBOT and anticancer therapy [3,16,61]. A further limitation is the frequent absence of direct or validated surrogate measurements of tumor oxygenation, making it difficult to determine whether inconsistent outcomes reflect biological inefficacy, inadequate reoxygenation, or suboptimal timing [9,10,12]. Human evidence is additionally weighted toward radiation-induced normal-tissue injury rather than direct tumor-control outcomes [3,16,61].

Future studies should treat HBOT as a measurable pharmacodynamic reoxygenation intervention and integrate baseline hypoxia with direct or surrogate measurements of oxygenation response. Relevant biomarkers may include HIF-regulated signaling, redox and metabolic state, vascular function, immune composition, and stromal organization [9,12,13,15,17]. Such measurements could identify tumor phenotypes most likely to respond to transient reoxygenation and enable biologically based patient stratification.

Treatment scheduling is likely to be critical because the biological effects of HBOT vary over time [16,61,68]. Future studies should define the magnitude and duration of tumor reoxygenation and synchronize anticancer treatment with this measurable window [16,61,68]. The goal should therefore not be to establish HBOT as a broadly applicable anticancer therapy but to determine whether biomarker-defined tumors exhibit a reproducible period in which reoxygenation reduces treatment resistance and increases therapeutic susceptibility.

Several limitations of the present review should also be acknowledged. This article was designed as a narrative rather than a systematic review and was intended to integrate mechanistic, preclinical, translational, and clinical evidence relevant to the context-dependent effects of HBOT in oncology. Literature was selected according to its relevance to the principal themes addressed in the review, including tumor hypoxia and reoxygenation, HIF signaling, redox and metabolic regulation, vascular and immune responses, cancer stemness, treatment sensitization, and radiation-induced tissue injury. Accordingly, the review did not employ a prospectively registered systematic-review protocol, exhaustive database-based study identification, formal risk-of-bias assessment, or quantitative evidence synthesis. The selection and interpretation of studies may therefore be subject to selection bias, particularly because the HBOT literature spans heterogeneous tumor models, treatment protocols, mechanistic studies, and clinical indications. In addition, greater emphasis was placed on studies providing mechanistic or clinically interpretable information, which may have resulted in underrepresentation of negative or less extensively characterized findings. The conclusions of this review should therefore be interpreted as a critical narrative synthesis intended to identify biological patterns, inconsistencies, and research priorities rather than as a formal estimate of treatment efficacy or safety.

## 6. Conclusions

Hyperbaric oxygen therapy occupies a context-dependent position in oncology. Overall, the available evidence does not support the assumption that HBOT universally promotes tumor growth, recurrence, or metastasis, while current findings are also insufficient to establish HBOT as a broadly applicable anticancer treatment. Its biological effects appear to vary according to tumor characteristics, including baseline hypoxia, genetic and metabolic phenotype, redox capacity, vascular and stromal organization, and treatment timing. Accordingly, the oncological consequences of HBOT should be interpreted within the specific biological and therapeutic context of each tumor rather than generalized across malignancies.

At present, the most established role of HBOT in oncology is the management of selected radiation-induced normal-tissue injuries, particularly late radiation cystitis. Its potential direct oncological application may be more appropriately explored as a biomarker- and timing-guided adjunct, in which treatment-induced reoxygenation is coordinated with anticancer therapy. Clarifying this potential role will require standardized HBOT protocols, objective assessment of tumor oxygenation, integration of mechanistic biomarkers, and prospective evaluation of treatment timing and patient selection.

## Figures and Tables

**Table 1:** Comparative Preclinical and Clinical Evidence for HBOT Across Oncological Contexts.

Cancer/Clinical Context	Model/Population	HBOT Protocol	Combination/Timing	Main Findings	Overall Interpretation	Evidence Type	Reference
Prostate cancer	LNCaP and PC-3 xenograft models	NR	HBOT alone	Repeated HBOT did not produce sustained acceleration of tumor growth or angiogenic activity	No consistent tumor-promoting signal in vivo	Preclinical, in vivo	[34,38]
Prostate cancer	Latent prostate cancer model	NR	HBOT alone	No evidence of accelerated latent tumor progression	No clear tumor-reactivation signal in this model	Preclinical, in vivo	[39]
Prostate cancer	DU-145, PC-3, and LNCaP cells	NR	HBOT ± cytotoxic agents	Altered proliferation and cell-cycle distribution; increased sensitivity to paclitaxel and, to a lesser extent, doxorubicin; minimal effect on cisplatin response	Effects on proliferation and chemosensitivity are phenotype- and drug-dependent	Preclinical, in vitro	[40,41]
Lung cancer/NSCLC	A549 xenograft model	2.5 ATA; 90 min/session; once daily, 5 days/week for 2 weeks	HBOT alone	Improved tumor oxygenation; reduced tumor growth; increased apoptosis despite increased CD31-positive vascular structures	Tumor suppression can coexist with vascular-marker increases	Preclinical, in vivo	[44]
Lung cancer/NSCLC	Hypoxic A549 and H1299 models	NR	HBOT alone	Attenuation of HIF-1α/PFKP signaling, glycolytic metabolism, hyperproliferation, and EMT	Reoxygenation may disrupt hypoxia-dependent metabolic adaptation	Preclinical, mechanistic	[45,46]
Lung cancer	CoCl_2_-induced hypoxic A549 cells	NR	HBOT following chemically induced hypoxia	Reduced migration, invasion, EMT-associated changes, and apoptosis resistance	Reversal of selected hypoxia-adaptive malignant phenotypes	Preclinical, in vitro	[47]
Colorectal cancer	Murine colorectal liver-metastasis model	2.4 ATA; 90 min/session; daily	HBOT alone	No sustained increase in metastatic tumor volume or adverse microvascular progression	No clear tumor-promoting effect in this model	Preclinical, in vivo	[49]
Colorectal cancer	Chemically induced colorectal neoplasia in mice	Reported chamber pressure 2.0265 bar; 60 min at treatment pressure; 15 min compression and 15 min decompression; every 24 h for 10 sessions	HBOT initiated at week 12; 5-FU 100 mg/kg i.p. once weekly during weeks 12–13	Increased apoptosis and reduced HIF-1α and CD44 compared with chemotherapy alone	Potential chemosensitization through modulation of hypoxia- and stemness-associated pathways	Preclinical, in vivo	[50]
Colorectal liver metastasis	Experimental metastasis model	NR	HBOT + SMA–pirarubicin micelles	Combination associated with greater antitumor activity	Potential adjunctive effect involving drug delivery and/or redox modulation	Preclinical, in vivo	[51]
Colorectal carcinogenesis	CRC cell and xenograft models	100% O_2_; 2.4 ATA; 90 min/session	HBOT alone or with melatonin	Increased apoptosis; reduced proliferation and modulation of HIF-, metabolic-, stemness-, and EMT-related pathways	Pleiotropic modulation of hypoxia-associated tumor biology	Preclinical, in vitro/in vivo	[52]
Breast cancer	MDA-MB-231 and BT-474 xenografts	NR	HBOT alone ± 5-FU	Reduced tumor growth and proliferative activity; reduced metastatic burden and EMT-associated markers in MDA-MB-231 tumors; no additional benefit with 5-FU	Potential oxygen-dependent antitumor effect, but chemosensitization is not universal	Preclinical, in vivo	[35]
Breast cancer—late radiation toxicity	Patients after breast irradiation	Protocol varied by study	HBOT for radiation injury	Improvements reported in pain, breast/arm symptoms, swelling, functional complaints, and quality-of-life domains	Supportive-care benefit reported, but effects vary by endpoint and study design	Clinical, prospective/observational	[53]
Breast cancer—late radiation toxicity	>1300 patients across included studies	Heterogeneous across studies	HBOT for radiation injury	Potential improvement in pain, fibrosis, lymphedema, and mobility impairment	Potential benefit, but certainty limited by heterogeneity and risk of bias	Systematic review	[55]
Breast cancer—late radiation toxicity	Women with late local toxicity after breast irradiation	2.5 ATA; four 20 min oxygen periods/session; 120 min total chamber session; 30–40 sessions over 6–8 weeks	HBOT treatment strategy	ITT pain endpoint not significantly improved; fibrosis improved; treatment completers showed improvement in pain and fibrosis	Randomized evidence supports outcome-specific rather than uniform benefit	Randomized clinical trial	[56]
Glioblastoma	In vitro and in vivo GBM models	NR	HBOT with chemotherapy and/or radiotherapy	Reduced stemness and increased sensitivity to temozolomide and radiotherapy	Treatment-sensitizing effect in selected models	Preclinical, in vitro/in vivo	[63]
Glioblastoma	Patient-derived GBM cells and microglia	1.9 or 2.5 ATA; 60 min/session	Radiotherapy administered within 30 min after high-pressure oxygen exposure	Reduced HIF-1α; altered glucose metabolism; increased ROS and radiosensitivity; relative microglial radioprotection	Differential responses of malignant and non-malignant brain cells to hyperoxic exposure	Translational, in vitro/3D	[64]
Glioblastoma	GBM/macrophage experimental models	NR	HBOT	Shift away from an M2-like toward an M1-like macrophage phenotype; reduced tumor-cell viability and increased apoptosis	Potential immunomodulatory effect	Preclinical	[65]
Glioblastoma/glioma	Patients receiving multimodal therapy	NR	HBOT followed by IMRT boost with chemotherapy	Feasible with mild toxicity; median OS 22.1 months and 2-year OS/PFS of 46.5%/35.4% reported	Clinical signal is hypothesis-generating; independent HBOT effect cannot be isolated	Clinical, non-definitive	[68]
Glioma	Intracranial GL261 model	NR	HBOT alone	Increased tumor growth, Ki-67, CD34, and microvessel density; reduced apoptosis	Cautionary tumor-growth signal in a selected model	Preclinical, in vivo	[36]
Glioblastoma	Experimental GBM models	NR	HBOT ± temozolomide	Increased proliferation occurred concurrently with suppression of HIF-1α/HIF-2α–Sox2 signaling, reduced stemness, and increased chemosensitivity	Proliferation and therapeutic sensitization may coexist	Preclinical, mechanistic	[37,69]
Radiation cystitis	Patients after pelvic radiotherapy	100% O_2_; 2.2 atm; 90 min/session; planned 40 sessions	HBOT supportive therapy	Complete or partial improvement in radiation-associated hematuria	Consistent supportive-care signal	Clinical, retrospective	[71]
Hemorrhagic radiation cystitis	602 patients across studies	Heterogeneous across studies	HBOT for late radiation injury	Partial or complete resolution in 84%; recurrence in 14% of patients with available follow-up	Favorable aggregate clinical response	Scoping review and meta-analysis	[72]
Hemorrhagic radiation cystitis	556 patients across studies	Heterogeneous across studies	HBOT for late radiation injury	Approximately 90% symptomatic improvement and 55% complete remission of hematuria	Clinically meaningful aggregate efficacy signal	Systematic review and meta-analysis	[73]
Late radiation cystitis	RICH-ART population	100% O_2_; 240–250 kPa (~2.4–2.5 ATA); 80–90 min/session; 30–40 sessions	HBOT vs. standard care	Sustained improvement in patient-reported urinary symptoms; generally well tolerated	Strongest randomized clinical evidence for an oncological supportive-care indication	Randomized phase 2–3 trial/long-term follow-up	[74,75]
Radiation cystitis	Multicenter registry population	No single standardized protocol verified across participating centers	HBOT supportive therapy	Improvements in hematuria, urinary distress, and patient-reported quality of life	Real-world clinical support for radiation-injury management	Multicenter registry	[76]

Abbreviations: 5-FU, 5-fluorouracil; ATA, atmospheres absolute; EMT, epithelial–mesenchymal transition; GBM, glioblastoma; HBOT, hyperbaric oxygen therapy; HIF, hypoxia-inducible factor; IMRT, intensity-modulated radiotherapy; NSCLC, non-small cell lung cancer; OS, overall survival; PFS, progression-free survival; ROS, reactive oxygen species. NR, exact HBOT pressure, exposure duration, or session schedule was not reliably reported or could not be independently verified from the primary study. No protocol values were inferred from general HBOT practice or secondary sources. Evidence-type descriptors refer to study design and do not represent a formal evidence-grading system.

## Data Availability

No new data were created or analyzed in this study. Data sharing is not applicable to this article.

## Abbreviations

The following abbreviations are used in this manuscript:

ATP	Adenosine triphosphate
EMT	Epithelial–mesenchymal transition
GBM	Glioblastoma
HBOT	Hyperbaric oxygen therapy
HIF	Hypoxia-inducible factor
IMRT	Intensity-modulated radiotherapy
LDHA	Lactate dehydrogenase A
MRI	Magnetic resonance imaging
NRF2	Nuclear factor erythroid 2-related factor 2
NSCLC	Non-small cell lung cancer
PDK	Pyruvate dehydrogenase kinase
PET	Positron emission tomography
ROS	Reactive oxygen species
VEGF	Vascular endothelial growth factor
